# Drug target prediction and prioritization: using orthology to predict essentiality in parasite genomes

**DOI:** 10.1186/1471-2164-11-222

**Published:** 2010-04-03

**Authors:** Maria A Doyle, Robin B Gasser, Ben J Woodcroft, Ross S Hall, Stuart A Ralph

**Affiliations:** 1 Department of Biochemistry & Molecular Biology, Bio21 Molecular Science and Biotechnology Institute, The University of Melbourne, Victoria, 3010, Australia; 2 Department of Veterinary Science, The University of Melbourne, 250 Princes Highway, Werribee, Victoria, 3030, Australia; 3 Current address: Peter MacCallum Cancer Centre, St Andrew's Place, East Melbourne Victoria, 3002, Australia

## Abstract

**Background:**

New drug targets are urgently needed for parasites of socio-economic importance. Genes that are essential for parasite survival are highly desirable targets, but information on these genes is lacking, as gene knockouts or knockdowns are difficult to perform in many species of parasites. We examined the applicability of large-scale essentiality information from four model eukaryotes, *Caenorhabditis elegans, Drosophila melanogaster, Mus musculus *and *Saccharomyces cerevisiae*, to discover essential genes in each of their genomes. Parasite genes that lack orthologues in their host are desirable as selective targets, so we also examined prediction of essential genes within this subset.

**Results:**

Cross-species analyses showed that the evolutionary conservation of genes and the presence of essential orthologues are each strong predictors of essentiality in eukaryotes. Absence of paralogues was also found to be a general predictor of increased relative essentiality. By combining several orthology and essentiality criteria one can select gene sets with up to a five-fold enrichment in essential genes compared with a random selection. We show how quantitative application of such criteria can be used to predict a ranked list of potential drug targets from *Ancylostoma caninum *and *Haemonchus contortus *- two blood-feeding strongylid nematodes, for which there are presently limited sequence data but no functional genomic tools.

**Conclusions:**

The present study demonstrates the utility of using orthology information from multiple, diverse eukaryotes to predict essential genes. The data also emphasize the challenge of identifying essential genes among those in a parasite that are absent from its host.

## Background

Until recently, the search for novel drugs against parasites has been carried out mainly using approaches that directly screen for inhibition of parasite growth or lethality. The current industry and regulatory focus on target-based drug development has meant that the search for new anti-parasitic compounds has also moved to a target-based paradigm. The completion of genome projects and large-scale expressed sequence tag (EST) surveys for a range of parasites now means that tens of thousands of potential drug targets are potentially accessible for many of these organisms. The major challenge now is not only to identify putative targets, but also to prioritize them, such that available resources can be focused on those most likely to lead to effective treatments/drugs. This aspect is most pressing for neglected infectious diseases which cause a disproportionate burden in developing countries and for which the costs of the drug development process have deterred investment by the pharmaceutical industry [1]. Thus, well-considered approaches to predict the most promising targets and to identify those most likely to be essential are required to increase the likelihood that lead compounds proceed to commercial development [2].

Identifying essential genes of pathogens is important because chemical inhibition of non-essential genes is unlikely to result in the death of the infectious agent, whereas the use of non-essential molecules, for example, for vaccine targets might rapidly select for mutants that evade protective immune responses. Essentiality, however, is difficult to define because its application is necessarily restricted to the experimental conditions being tested. Despite this limitation, indispensability under a defined laboratory condition can be a useful starting point for identifying biological processes that are likely to be crucial for the survival of an organism under natural conditions. The proliferation of genome sequencing projects has been followed by systematic analyses of gene knockouts and/or knockdowns to determine essentiality in a number of model eubacteria and eukaryotes, such as *Escherichia coli*, *Saccharomyces cerevisiae *and *Caenorhabditis elegans *[3-8]. Unfortunately, few infectious agents are currently amenable to whole-genome approaches for the experimental testing of essentiality, such that promising candidate drug targets must be individually validated genetically. This is particularly true of eukaryotic parasites, for which the genetic tools to characterize drug or vaccine targets are often limited, and absent for many others.

A particularly challenging area is the development of novel compounds to combat parasitic helminths of animals. Such helminths are of major socio-economic importance; more than a billion people are infected with soil-transmitted worms (= geohelminths), such as the blood-feeding hookworms *Ancylostoma duodenale *and *Necator americanus*, the common roundworm, *Ascaris lumbricoides*, and the whipworm, *Trichuris trichiura *[9,10]. These parasites alone impose a worldwide annual burden of 39 million Disability Adjusted Life Years (DALYs) and cause serious adverse effects on human health, particularly in children [10]. Similarly, parasitic nematodes of livestock, such as cattle and sheep, also cause substantial economic losses due to the diseases they cause, with billions of dollars spent annually on the treatment of gastro-intestinal nematodes alone. Resistance is emerging to all of the main classes of anthelmintics used to combat parasitism [11-14], creating a serious need for new classes of compounds.

Although desirable, anthelmintic targets do not necessarily need to be encoded by essential genes. For example a number of anthelmintics (such as aldicarb and trichlorfon) target acetylcholinesterases, and perturbations or silencing of *C. elegans *acetylcholinesterase genes can result in a viable phenotye (but sometimes with drug resistant phenotypes) [15]. Likewise, levamisole targets acetylcholine receptors, and the disruption of several subunits of these receptors also leads to a viable levamisole resistant phenotype[16]. Despite the precedence for successful drugs that act through agonist relationships with non-essential molecules, essentiality is a desirable character of genes to be pursued by a target-centric approach. Although the relative success of target-based screening is uncertain compared with whole organisms screens, future screening against single molecule targets is likely to measure loss-of-function linked to lethality, rather than searching for modulators or agonists. Therefore, the prediction and identification of essential genes in parasitic helminths is considered an important step towards the prioritization of drug targets in nematodes.

There has been significant growth in the amount of sequence data for nematodes, enabling the prediction of numerous potential drug targets. Much focus has been on the sequencing of ESTs [17-23] due, in part, to difficulties in producing sufficient quantities of genetically homogenous material from heterologous worm populations for genomic sequencing. However, some progress has been made on whole genome sequencing (e.g., *Brugia malayi*) [24], and a program has recently received funding for the sequencing of a range of socio-economically important nematodes of the order Strongylida [25]. The data emerging from these sequencing projects have spurred the identification of drug targets in parasitic nematodes using bioinformatic techniques (e.g., [26]), but the testing of essentiality for these targets has generally been absent. Members of the Strongylida cannot be propagated (through the entire life cycle) in culture *in vitro*. In addition, gene silencing (double-stranded RNA interference, RNAi) does not work effectively in the parasitic nematodes of this order assessed thus far, such that, currently, there are major limitations in conducting functional studies in different developmental stages of these parasites [27-29] in order to pre-validate potential drug targets.

The paucity of experimental tools to characterize drug targets in the Strongylida intensifies the need to predict which putative targets are most likely to be essential; in this case, predictions are necessarily based on inference from orthologues in model organisms. However, a rational method for applying knowledge of essentiality from orthologues in other species is lacking. Taking advantage of recent advances in whole genome orthology mapping [30] and the availability of large-scale eukaryotic essentiality datasets [31-33], we have created a database to analyse how essentiality can be predicted from existing data from model eukaryotes. The designation of essentiality in these diverse organisms means quite different things. In yeast, essentiality can refer to a failure to grow in culture, whereas in mice it can encompass the failure of embryos to progress to live offspring.

In the present study, we tested whether these disparate forms of essentiality are predictive of the corresponding phenotypes in other organisms and show that definitions of essentiality are strongly predictive of essentiality even in relatively distantly related eukaryotes. We applied lessons from these analyses to the prediction of essential genes in parasitic nematodes. Members of the order Strongylida are considered to be relatively closely related to *Caenorhabditis elegans*, being within clade V [34], such that it is considered appropriate to infer gene function from the abundant functional genomic data (including a whole genome RNAi knockdown) that exist for *C. elegans *[26,35,36]. In addition to a rational prediction of essentiality from orthology and essentiality data, we integrated recently developed gene network connectivity data [37] to further predict key, essential genes that may offer promising and testable drug targets.

Another criterion that can be employed for the prediction of drug targets in infectious organisms is the absence of a corresponding host orthologue, so as to maximise opportunities for pharmacological selectivity. However, many successful exceptions to this criterion exist; for example, genuine targets may be differentially regulated between pathogen and host [38], protein turnover may be more rapid in the host than the pathogen [39], targets may be active only in proliferating and cancerous cells in the host [40], or may be sufficiently structurally different between host and pathogen to allow selectivity [41]. We investigated the effect of excluding genes that possess mammalian orthologues and discovered that the remaining genes are much less likely to be essential. Therefore, we developed more flexible prioritization methods that are not limited by the requirement for genes to lack host orthologues. In the present study, we applied these techniques to predict of essential drug targets in large EST data sets representing two blood-feeding strongylid nematodes of major socio-economic importance, namely *Haemonchus contortus *(the barber's pole worm of small ruminants) and *Ancylostoma caninum *(canine hookworm). We combined this prioritization strategy with other criteria to predict molecules as drug targets in these haematophagous nematodes as a proof-of-principle application of using orthology to predict essentiality.

## Results

### Database of essentiality

**Figure 1:**
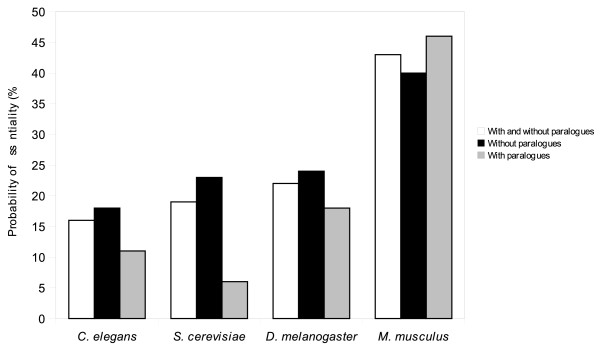
**Effect of paralogy on probability of essentiality of genes**. This graph shows the probability of essentiality for genes with and without paralogues for *C. elegans*, *D. melanogaster, M. musculus *and *S. cerevisiae*. Presence of paralogues means more than one gene from the species is present in an OrthoMCL group. Percentages are numbers of genes with lethal phenotypes expressed as a fraction of genes with available phenotype information for each species. Differences in essentiality of genes with versus without paralogues were highly statistically significant (P < 10^-04^), as determined using Fisher's exact test (Additional File 2).

To enable the comparison of phenotypes among the four model organisms - *Caenorhabditis elegans, Drosophila melanogaster, Mus musculus *and *Saccharomyces cerevisiae - *a database was constructed to allow the mapping of phenotype data among orthologues. Orthology was determined using the OrthoMCL database [30], and was populated with phenotype data for these four species. In the case of *C. elegans*, *D. melanogaster *and *S. cerevisiae*, phenotype information was available for 94%, 75% and 88% of all protein-coding genes respectively. For *M. musculus*, gene knockouts/knockdowns represented 20% of all protein-coding genes. Because the genes chosen for knockout analysis in mice were selected to address specific phenotypic questions, it is possible that this non-random collection may introduce some bias towards or away from genes that are expected to be essential. Indeed, genetic experiments involving mouse genes did have a higher percentage of reports of lethality/essentiality than the other organisms studied (Figure 1), which may reflect selection bias. Nonetheless, the patterns observed for the prediction of essentiality from orthology in the *M. musculus *dataset paralleled those in the other organisms assessed, supporting their inclusion in the present analyses.

### Prediction of essentiality based on phyletic characteristics - presence of paralogues

We interrogated our database to determine whether paralogy was a useful predictor of essentiality. Intuitively, belonging to a paralogous family may suggest some degree of redundancy that could compensate for a disrupted gene; indeed, previous studies of *C. elegans *and *S. cerevisiae *have indicated that genes with paralogues are less likely to be essential [42,43]. However, in the mouse, a recent analysis [44] revealed no difference between genes with and without paralogues. These studies used different criteria to define paralogues and, in some cases, included genes that cause sterility as being essential, making it difficult to compare results among studies. Therefore, to compare the effect of paralogy on the likelihood of lethality among different species, we used a single method for the definition of a paralogue (OrthoMCL), combined with existing large-scale essentiality data for *C. elegans*, *D. melanogaster, M. musculus *and *S. cerevisiae*. The presence of paralogues was inferred from OrthoMCL groups; if there was more than one gene from a species in an OrthoMCL group, these genes were classified as genes with paralogues. The probability of essentiality for genes with or without paralogues is shown in Figure 1 and Additional File 1: Table S1.

For *C. elegans *and *S. cerevisiae*, we found that genes without paralogues were significantly more likely to be essential than genes with paralogues (Figure 1). For *C. elegans*, there was a 1.6-fold greater probability of essentiality for genes without paralogues (P < 10-^33^, Fisher's exact test, Additional File 2), whereas for *S. cerevisiae*, there was a 3.8-fold greater probability of essentiality (P < 10-^51^). For *D. melanogaster*, genes without paralogues were also more likely to be essential (1.4-fold, P < 10-^12^), though to a lesser extent than for *C. elegans *and *S. cerevisiae*. The incomplete dataset of *M. musculus *differed from those of the other three species in that genes without paralogues were (0.9-fold, P < 10-^04^) less likely to be essential than genes with paralogues.

### Prediction of essentiality based on phyletic characteristics - presence of orthologues

**Figure 2:**
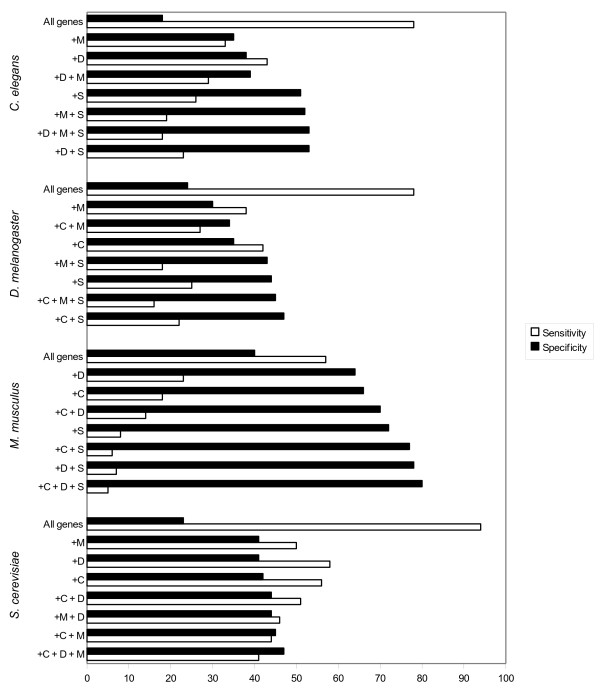
**Effect of gene conservation on probability of essentiality of genes**. This graph shows the probability of essentiality for genes from *C. elegans*, *D. melanogaster*, *M. musculus *and *S. cerevisiae *respectively, modified by their phyletic profile. The percentages show the number of genes with lethal phenotypes divided by the total number of genes with orthologues in the species being compared. Specificity refers to the proportion of genes in the selected subset that are essential and sensitivity refers to the proportion of the total number of essential genes for that species that are recovered in the selected subset. In all cases, essentiality was assessed for genes without paralogues. The probability of essentiality for genes in each of the specified subsets versus a gene selected at random from the query genome were all statistically significant (P < 10^-05^), as determined using Fisher's exact test (Additional File 2). C = *C. elegans*, D = *D. melanogaster*, M = *M. musculus*, D = *D. melanogaster*, S = *S. cerevisiae*.

Genes that are shared among species have been hypothesized previously to be those that are more likely to be essential [45]. However, there is no evidence that this proposal has been tested quantitatively for eukaryotes, and we were unaware of published studies that demonstrated the effect of the breadth and extent of conservation over large evolutionary distances on the probability of essentiality. Indications that gene conservation could be used as a predictor of essentiality in eukaryotes comes from a study of 315 essential genes of *Danio rerio *(zebrafish) [46], which found that essential genes were more likely to be conserved than non-essential genes. To test the effect of conservation of genes on essentiality, we analysed the frequency of essentiality among genes with orthologues in *C. elegans*, *D. melanogaster, M. musculus *and *S. cerevisiae*. For each of the four species, we tested the effect of conservation of a gene in one or more species on essentiality in another (query) species. For example, to examine how well the presence of an orthologue in *S. cerevisiae *predicts essentiality in *C. elegans*, we first identified orthology groups comprising genes from these two species, and then, for the *C. elegans *genes in these groups, we calculated the percent that were essential. The findings are shown in Figure 2 and Additional File 1: Table S2.

For genes in each of the four species, the existence of an orthologue in other species increased the probability of essentiality, and the more distant the source of the orthologue, the greater this effect. For *C. elegans*, the probability of essentiality for a gene without paralogues selected at random was 18%; this probability increased to 38% if the gene had an orthologue in *D. melanogaster *and to 51% if an orthologue existed in the more distant relative, *S. cerevisiae *(Figure 2). Conservation over a greater evolutionary distance therefore appears to be a better indicator of essentiality than conservation between or among organisms that have diverged more recently. We also tested whether the conservation of orthologues among multiple species would improve the probability of essentiality in any particular species. For nearly all combinations, this was indeed the case (see Figure 2). For example, 51% of *C. elegans *genes with orthologues in *S. cerevisiae *are essential, and 53% of *C. elegans *genes that have orthologues in both *S. cerevisiae *and *D. melanogaster *are essential. There are several exceptions whose causes are unclear- e.g., genes present in all four species, *C. elegans*, *D. melanogaster, S. cerevisiae and M. musculus*, were less likely to be essential when knocked down in *Drosophila *than those that were present in only the former three species (Figure 2).

### Prediction based on orthology mapping of existing essentiality data for the four species

**Figure 3:**
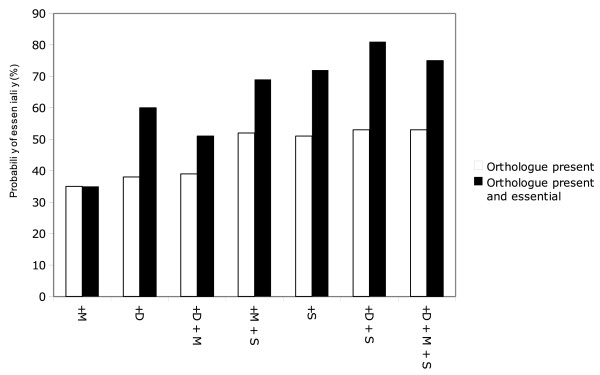
**Effect of conservation of essential genes on probability of essentiality**. This graph shows the likelihood of essentiality for *C. elegans *genes with essential orthologues in one or more of the organisms; *D. melanogaster, M. musculus *and *S. cerevisiae*. The percentages are the number of genes with lethal phenotypes divided by the total number of genes with orthologues in that species. Essentiality was assessed for genes without paralogues. C = *C. elegans*, D = *D. melanogaster*, M = *M. musculus*, S = *S. cerevisiae*.

Subsequently, we tested whether the presence of an essential orthologue is more predictive of the essentiality of a gene than the presence of a non-essential orthologue. This has been explored previously in the bacterium *Pseudomonas aeruginosa*, for which essential genes have been predicted by identifying genes that are homologous to essential genes in other unicellular micro-organisms, such as *Mycoplasma genitalium*, *Haemophilus influenzae*, *Vibrio cholerae*, *Staphylococcus aureus*, *E. coli *and *S. cerevisiae *[47]. This set of genes predicted to be essential was indeed in agreement with genes shown experimentally to be essential. However, whether essentiality of an orthologue can be used to predict essentiality for multi-cellular organisms had not been tested. For *C. elegans*, *D. melanogaster, M. musculus *and *S. cerevisiae*, we assessed whether essentiality in one species could be used as a predictor of essentiality in a second species. To do this for each query species (ie *C. elegans*, *D. melanogaster, M. musculus *and *S. cerevisiae*), we assessed the probability of essentiality for genes that have lethal phenotypes in the other three species. When comparing phenotypes linked to particular genes among species, we limited the analysis to genes with no paralogues in each of the species being compared; this was conducted to avoid the difficulty of comparing phenotypes when individual paralogues were associated with different phenotypes. To test whether having essential orthologues in *S. cerevisiae *predicted essentiality in the corresponding *C. elegans *genes, we identified orthology groups containing non-paralogous *C. elegans *and *S. cerevisiae *genes that had been subjected to essentiality analysis. We then compared the frequency of essentiality for *C. elegans *genes that grouped with essential or non-essential *S. cerevisiae *orthologues. We also tested whether essentiality in multiple species improved the probability of essentiality. The results of the predictions for *C. elegans *and the other three species are shown in Figure 3 and Additional File 1: Table S3, respectively.

Our analysis showed that a gene with an orthologue with a lethal knockout/knockdown phenotype was more likely to be essential than if the orthologue were non-essential; the more distant the orthologue, the greater was the probability of essentiality. Thus, the existence of an orthologue associated with a lethal phenotype in *S. cerevisiae *was highly predictive of lethality in *C. elegans *(72%), greater than the presence alone of an orthologue in *S. cerevisiae *(51%). Combining the presence of an essential orthologue in an evolutionarily more distant relative (*S. cerevisiae) *with an additional, closely-related species (i.e., *D. melanogaster) *increased this probability further - *C. elegans *genes with an orthologue in both *D. melanogaster *and *S. cerevisiae*, each with a lethal phenotype, had an 81% probability of lethality.

### The effect of drug-target selectivity on predictions of essentiality

Anti-parasitic drugs need to selectively inhibit or kill the parasite without causing unacceptable toxicity to the host. One way of pursuing targets for such drugs is to focus on parasite genes that lack host orthologues. As our aim was to identify targets for parasitic nematodes that infect mammals, we examined the possible effects on essentiality of focusing on parasite genes that lack mammalian orthologues. To do this, we analysed the probability of essentiality for *C. elegans *genes that either possessed or lacked mammalian orthologues. To obtain the set of *C. elegans *genes without mammalian orthologues, we identified *C. elegans *genes that were in an OrthoMCL group that did not have a gene in any of the three mammals considered (i.e., *Homo sapiens*, *M. musculus *and *Rattus norvegicus*). We then calculated the probability of essentiality of these genes. *C. elegans *genes that lacked mammalian orthologues were much less likely to be essential; the probability of essentiality decreased from 16% to 8% for a gene selected at random, and was only 6% if the gene had paralogues (Table 1, Figure 3). This substantial decrease in the probability of essentiality has important consequences for drug target prediction that have not been made explicit previously. Researchers are quite likely to examine genes in exactly this category (i.e. present in a pathogen but absent from the host), but the very low "raw" probability of essentiality for these genes (6-8%) highlights the challenge for the prediction of essential drug targets from such a subset, which we address here.

**Table 1:** Combining essentiality with selectivity

	Probability of essentiality		
	***Genes with and without paralogues***	***With paralogues***	***Without paralogues***

*C. elegans* (all genes)	**16%** (2941/18860 genes)	**11%** (656/5988 genes)	**18%** (2285/12872 genes)

*C. elegans *(excluding genes with mammalian orthologues)	**8%** (1090/13307 genes)	**6%** (249/4431 genes)	**9%** (841/8876 genes)

This table demonstrates the effect of excluding genes with mammalian orthologues on the probability of essentiality. Percentages shown are the number of genes with lethal RNAi (gene knock-down) phenotypes divided by the total number of genes in each category. The numbers of genes are given in brackets.

### Prediction of drug targets in *Anyclostoma caninum *and *Haemonchus contortus*: combining 'assayability' with the prediction of essentiality

**Figure 4:**
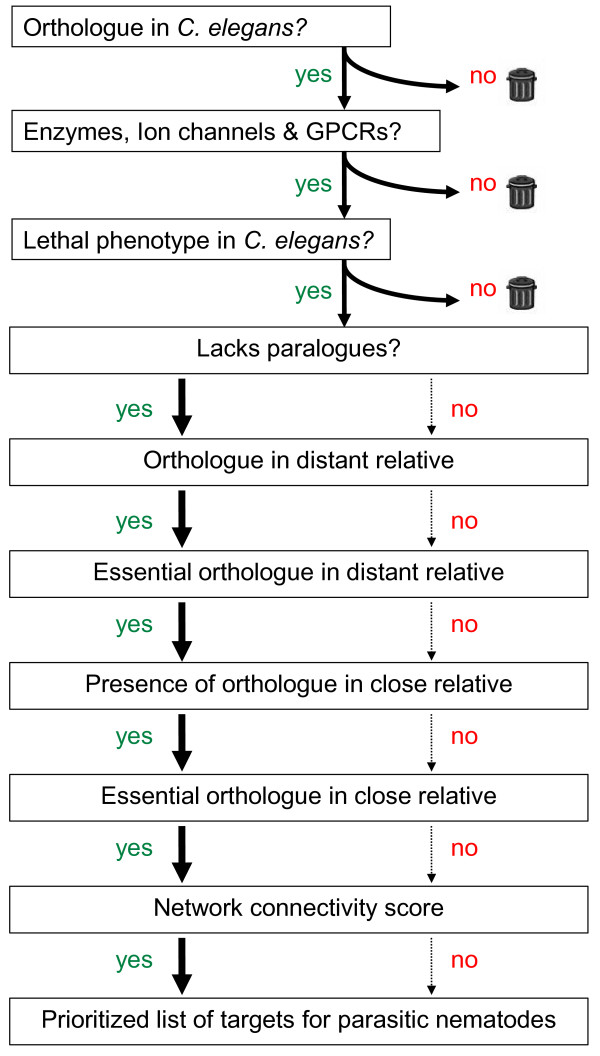
**Prioritizing essential nematode drug targets**. This flow diagram outlines our approach to drug target prediction and prioritization for parasitic nematodes. Some steps are clear "keep or reject" decisions because the genes failing these criteria are either not easily amenable to further development or have an unacceptable probability of being essential. Other steps are used to quantitatively refine the weighting to produce a ranked list of essential drug targets.

The present findings were applied to the prediction of drug targets in haematophagous parasitic nematodes, as outlined in a flow diagram (Figure 4). The parasites *Ancylostoma caninum *and *Haemonchus contortus *were selected to represent parasitic nematodes of the order Strongylida ("clade V" [34]), for which significant EST sequence data exist. All available EST data for *A. caninum *and *H. contortus *(comprising 21,975 and 80,551 ESTs, respectively) were downloaded from the NCBI database. These ESTs were first mapped to the OrthoMCL orthology groups by a BLASTX analysis of individual EST sequences against all OrthoMCL proteins using the OrthoSelect method [48]. A caveat of this EST-based analysis is that the incompleteness of genomes for the parasitic nematodes means that there will be poorer identification of paralogues and orthologues. This analysis was followed by the extraction of *C. elegans *orthologues from the orthology groups. Previously, we showed that having an essential orthologue was a better predictor of essentiality than just having an orthologue (e.g., for *C. elegans*, a gene that has an orthologue that is essential in *D. melanogaster *is 1.6-fold more likely to be essential than if the *C. elegans *gene just has an orthologue in *D. melanogaster*). We therefore focused on the subset of *C. elegans *orthologues of the parasitic nematodes that were essential (i.e., genes that have lethal RNAi phenotypes in *C. elegans*).

### Assayability requirement: enzymes, ion channels and GPCRs

To select targets that are most amenable to *in vitro *screening for growth inhibition or lethality, we focused on genes encoding proteins for which functional assays exist. Thus, we examined the subset of *C. elegans *genes encoding proteins inferred to have enzymatic, ion channel, or GPCR activity, as determined by GO term annotation (i.e., GO:0003824, GO:0005216 or GO:0004930, or a subterm thereof). All current drugs in clinical use against nematodes target a protein in one of these three categories. GPCRs are the targets of more than 50% of pharmaceutical drugs [49], making them attractive targets also in parasitic nematodes. We found that enzymes were more likely to be essential than non-enzymes for both *C. elegans *and *S. cerevisiae *- for *C. elegans*, 16% of all genes were essential, whereas 21% of enzymes were essential; for *S. cerevisiae*, 19% of genes were essential, while 24% of enzymes were essential - so, by focusing on enzymes, we further increased our likelihood of identifying essential genes.

### Essentiality requirement: quantitative formula for maximum prediction of essentiality based on the present analysis

We applied a quantitative weighting scheme to the genes that met the criterion for assayability, according to the present analysis of the relative importance of each feature in determining essentiality. For example, we showed that having no paralogue increased the probability of essentiality by 1.6-fold in *C. elegans *(Table 1), having an orthologue with no paralogues in *S. cerevisiae *or *D. melanogaster *increased probability by 3.3 and 2.4-fold, respectively. (Figure 3). Furthermore, having an orthologue with no paralogues that was essential in *S. cerevisiae *and *D. melanogaster *increased the probability of essentiality by 5.2-fold. These fold increases were all statistically significant (P < 0.0005), as determined using Fisher's exact test (Additional File 2). This weighting should thus score genes numerically in order of the probability that such genes are essential.

### Increasing the probability of essentiality by incorporating information on network connectivity

Recently, Lee et al. [37] constructed a probabilistic functional interaction network for *C. elegans*, the largest such network to date, comprising ~82% of all *C. elegans *genes. This network integrates data for proteins and genes (e.g., microarray data and interaction and association information) using information about *C. elegans *genes as well as their orthologues in other species. Using this network, the authors showed that gene connectivity is predictive of essentiality - genes that were highly connected in the network were more likely to be essential; this was shown to be a feature that was conserved across eukaryotes (including *S. cerevisiae *and *M. musculus*). An independent study by Hwang and colleagues also showed that genes encoding proteins with higher connectivity in protein-protein interaction networks were more likely to be essential in *E. coli *and in *S. cerevisiae *[50]. As genes with higher network connectivity scores (as assigned by Lee *et al*) were more likely to be essential than those with lower scores [37], we gave greater weighting to genes with higher connectivity scores.

We combined the quantitative weighting scheme with network connectivity scores to produce a prioritized list of putative drug targets for *A. caninum *and *H. contortus *(Additional file 3). These represent predicted *C. elegans *orthologues of the *A. caninum *and *H. contortus *ESTs that were chosen for assayability (enzyme, ion-channel or GPCR), with a quantitative ranking scheme applied to predict essentiality. Genes were included in the list if they related to a lethal RNAi phenotype in *C. elegans*.

Although the list comes from an incomplete EST snapshot of these haematophagous helminths, it nevertheless recovers three validated anthelmintic drug targets; beta-tubulin, target of Benzimidazole [51]; glutamate-gated chloride channel, a target of the avermectin/milbemycin anthelmintics [52] and succinate dehydrogenase/fumarate reductase, the *C. elegans *equivalent of one helminth target of thiabendazole [53].

**Figure 5:**
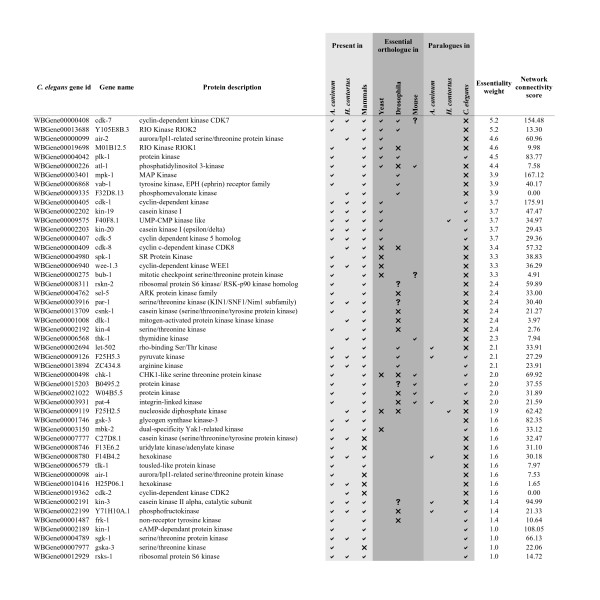
**Prioritized kinases for the parasitic nematodes *A. caninum *and *H. contortus***. This shows the *C. elegans *predicted orthologues of the *A. caninum *and *H. contortus *ESTs, as identified in the present study. All genes are predicted kinases, have a lethal RNAi phenotype in *C. elegans*. Targets were first prioritized by the essentiality weighting assigned from the present study, then by Wormnet connectivity score. The top two ranked kinases (in bold) are among the top thirty ranked targets from all ESTs (Additional File 3). Present in mammals is defined by having an orthologue in one or more of the mammalian species in the OrthoMCL 2.0 database (human, rat, mouse). Essential orthologue in yeast, drosophila or mouse: ✓ = orthologue (with no paralogues) associated with a lethal phenotype, ✘ = orthologue (with no paralogues) that had only non-lethal phenotypes recorded, **? **= orthologue (with no paralogues) but no phenotype information was available. Presence of paralogues in *C. elegans *was determined from the OrthoMCL grouping. Presence of paralogues in *A. caninum *and *H. contortus *was determined as described in Materials and methods. The network connectivity score is the sum of all interaction scores (log-likelihood scores) for the gene in the Wormnet core network [37].

A likely application of this prioritization method could be to focus on a particular subset of enzymes in which an investigator has expertise. For example, if the aim was to identify promising kinase drug targets, all kinases from the ranked list could be extracted and appraised. There are 49 predicted kinases (Figure 5) in the *H. contortus *and *A. caninum *EST datasets. Those at the top of the list would be expected to be most likely to be essential in these two parasitic nematodes, based on the present analyses. Although the numerical weighting cannot give an exact probability of essentiality for each uncharacterized gene, the weightings should give an estimation of the relative likelihood of being essential for any two genes being considered as candidate targets.

## Discussion

Defining essential genes is a central strategy in the prioritization of drug targets in infectious organisms. Although some non-essential genes may act as drug targets by converting non-toxic precursors to toxic products, most anti-microbials act by inhibiting the function of an essential process, leading to the death of the pathogen, either through direct absence of that essential process or, indirectly, though a stress-related response. A number of anthelmintic drugs (e.g. levamisole [16] and ivermectin [54]) appear to function as agonists of non-essential genes (for a review of anthelmintic modes of action see [55]). Although the search for drug targets among essential genes is a useful approach for the prioritization of targets, the inability of this methodology to predict some of the successful anti-parasitic drug targets is a limitation of this practice.

There have been several attempts to define or predict essential genes in infectious agents; in a few cases, where sophisticated functional genomic tools have been available, this has been possible through the systematic disruption of candidate genes, for example, in *Streptococcus pneumoniae *[56], *Candida albicans *[57], *Aspergillus fumigatus *[58]) and *Mycobacterium tuberculosis *[7,8]. However, for the vast majority of infectious organisms, high-throughput experimental interrogation of essentiality is currently not possible, and efforts to define essentiality have been based on bioinformatic predictions [59-63].

Here, we have attempted to define general characters that are predictive of essentiality of eukaryotic genes through orthology. Previous studies have shown that conservation between genomes is itself a predictive feature of essential genes. Studies by Butland, [64] Hwang [50] Krogan [65] and colleagues showed that essential genes in *S. cerevisiae *and *E. coli *are both more widely phyletically distributed, and are more connected through physical and functional protein-protein interactions. Our data show that broad evolutionary conservation of genes (among multicellular eukaryotes) is predictive of essentiality, and also that essentiality of those orthologues is a strong predictor. Combining individual criteria that are independently predictive of essentiality increases the specificity of prediction - for example, a *C. elegans *gene selected at random is only 16% likely to be essential, whereas *C. elegans *genes that lack paralogues and have essential orthologues in *S. cerevisiae *and *D. melanogaster *are 81% likely to be essential. This dramatic increase in the specificity of prediction increases the probability of essentiality from a dishearteningly low level, at which a randomly chosen drug target candidate is very likely to be non-essential, to a reassuring level where prioritized candidates are very likely to be essential. However, the increase in specificity associated with these predictions is also accompanied by a decrease in sensitivity - many genuinely essential genes are discarded in such highly prescriptive queries. In the present example, only 258 candidates remained from a set of 2941 *C. elegans *genes determined experimentally to be essential. In practice, the investigator must balance the desired specificity of the query against the desired pool size of the selected targets, or highly weight the most specific predictors, so that genes most likely to be essential are ranked at the top.

The discovery that the exclusion of genes with mammalian orthologues leads to sets of genes that are much less likely to be essential has major implications, because it highlights an additional challenge for the identification of drug targets that still allow selectivity. The decreased essentiality can be readily rationalised, considering the analyses herein of the relationship between orthology and essentiality. In this case, we excluded a set of genes that shared orthology between worms and mammals. The resultant set, conserved between fewer organisms, contained a higher proportion of non-essential genes.

We focused on enzymes, ion channels and G-protein-coupled receptors (GPCRs) because the vast majority of all drugs target these classes [49,66] and chemicals already exist to interrogate such targets. Furthermore, we found that the proportion of essential genes is greater for enzymes than for other protein-coding genes, for both *C. elegans *and *S. cerevisiae*.

In this study, we applied semi- quantitative methods to identify essential genes in parasitic helminths. These methods should be equally applicable to the prediction of essential genes in many eukaryotic pathogens with EST or genome projects. We have shown that genes that have essential orthologues have a greatly increased probability of essentiality so a researcher could identify the genes in their parasite of interest that were essential in *C. elegans*, *D. melanogaster*, *M. musculus *and/or *S. cerevisiae*. As these species are all distantly related to protozoan species, essentiality in one or more of these species could be highly suggestive of essentiality in a protozoan parasite *versus *genes that are not essential in any of these model organisms.

In addition to identifying drug targets, identification of promising vaccine candidates is also a priority for parasitic diseases. Although essentiality is likely to be an important characteristic of vaccine targets, a large variety of other characters are also desirable but currently difficult to establish bioinformatically, particularly for an incomplete genome. Although these characters may become more tractable in the near future, the current approach is not appropriate for identifying vaccine antigens.

## Conclusions

In this study, we undertook a quantitative assessment of essentiality and its inference using orthology. This analysis demonstrates the utility of examining multiple, diverse organisms when predicting essentiality, and shows that essentiality for one gene is highly predictive of essentiality for its orthologues, even for organisms with extremely different life cycles. These data also highlight the potential for undesirably enriching for non-essential genes when prioritizing genes in a pathogen that are absent from their host. We quantitatively applied the conclusions from this analysis to the prediction of essential genes in blood-feeding parasitic nematodes, combining this with the requirement for assayability to identify targets readily amenable to further investigation. Our analysis has led to the prioritization of a tractable number of potential drug targets leading toward the development of nematocidal agents.

## Methods

### Orthology data

Orthology data were obtained from OrthoMCL [30], available at http://www.orthomcl.org. OrthoMCL classifies genes from 87 genomes into orthology groups. Files containing protein sequence data and orthology group information were downloaded from OrthoMCL version 2. This dataset contained 20084, 14034, 24435 and 6718 proteins from *C. elegans, D. melanogaster, M. musculus *and *S. cerevisiae*, respectively and includes genes assigned to orthology groups in the OrthoMCL dataset and also those not assigned to any group (annotated in OrthoMCL with 'no_group'). Orthologues are genes deriving from an ancestral gene that were separated by a speciation event, while paralogues are genes that were separated by a gene duplication event within a genome. Subsequent speciation can lead to paralogous relationships between genes from different genomes (referred to as out-paralogues) [67]. In practice, the identification of these relationships is non-trivial and is most robust where complete genome data and sequences with useful phylogenetically informative characters allow unambiguous phylogenetic trees to be constructed. This is currently not computationally or practically feasible for multi-eukaryote, whole genome analyses. The OrthoMCL clustering method provides a convenient, but necessarily imperfect means of estimating orthology and paralogy. In-paralogues and out-paralogues that are separated by a recent duplication event will normally be clustered into a OrthoMCL group that may represent a gene/protein family, whereas more ancient duplications are more likely to result in separate clusters [68]. For the purposes of this paper, genes from different species that share the same OrthoMCL cluster are considered to be orthologues, while multiple genes from within the same species that share the same OrthoMCL cluster are considered paralogues. Additional File 4 demonstrates example phylogenetic trees of three representative types of orthologue clusters.

### Phenotype data

Phenotype information was obtained for *C. elegans*, *M. musculus *and *S. cerevisiae *from the databases Wormbase WS191 http://www.wormbase.org[69], Mouse Genome Informatics (http://www.informatics.jax.org; June 2008 [70]) and Saccharomyces Genome Database (http://www.yeastgenome.org; Aug 2008) [71], respectively. For *D. melanogaster *we used the results of the genome-wide RNAi screen [33]). In order to focus on phenotypes that result from disruption/inhibition of proteins, as opposed to overexpression phenotypes, we restricted our analysis to phenotypes from the following experiment/mutant types: *C. elegans *(RNAi), *S. cerevisiae *(null, reduction of function, repressible), *D. melanogaster *(RNAi) and *M. musculus *(included phenotypes from the following experiments: Chemically and radiation induced, Chemically induced (ENU), Chemically induced (other), Gene trapped, Radiation induced, Spontaneous, Targeted (knock-out), Targeted (Reporter), Targeted (Floxed/Flt), Transgenic (random, gene disruption)). Essential genes were defined as follows; for *C. elegans *and *M. musculus *these were genes with a phenotype description containing the term 'lethal' (see Additional File 5 for a list of these phenotypes), for *S. cerevisiae *these were genes with the phenotype 'inviable', for *D. melanogaster *these were genes that showed lethality during the Adult, Pupal, Before Pupal or Eclosion life-stage from [33]. We cannot exclude the possibility that some of the phenotypes from *M. musculus *may not result from protein disruption/inhibition, for example, it is possible that chemical or spontaneous mutation could result in constitutive activation of a protein or a gain-of-function. However, including these phenotypes should only result in a more conservative prediction of essentiality because it would mean that some of the *M. musculus *genes might be falsely classed as essential orthologues and when used to predict essentiality in *C. elegans *would lead to a lower probability of essentiality for *C. elegans*.

### Construction of a database for whole-genome comparisons of essentiality

To enable the comparison of phenotypes among all four species, a relational database was constructed to map phenotype data for these species to their OrthoMCL genes and groups. This database was a PostgreSQL database http://www.postgresql.org which used a Ruby on Rails framework http://www.rubyonrails.org. Custom tables were designed to store the data and the OrthoMCL proteins were mapped to the genes in the phenotype files using the gene identifiers present in the datasets (identifiers of the type WBGene00000001 for *C. elegans*, CG3095 for *D. melanogaster*, ENSMUSG00000020473 for *M. musculus *and YNL214W for *S. cerevisiae)*. The software used in this process is available at http://github.com/wwood/essentiality. Phenotype data were available for 94% (18860/20084), 75% (10572/14034), and 88% (5908/6718) of *C. elegans, D. melanogaster *and *S. cerevisiae *genes in the OrthoMCL dataset, respectively. In contrast, phenotype data were available for 20% (5008/24435) of *M. musculus *OrthoMCL genes.

### Computational predictions of essentiality

Custom Ruby scripts were written to query the database through the Ruby on Rails framework. Genes without paralogues were defined as those genes in OrthoMCL groups with only one gene from the query species, or genes from the query species in the OrthoMCL dataset that were assigned 'no_group'. The number of genes with paralogues was obtained by subtracting the number of genes without paralogues from the total number of genes for the query species.

To analyze the effect of *presence *of orthologues on probability of essentiality, for example, the effect of having a yeast orthologue on probability of essentiality of *C. elegans *genes, the OrthoMCL groups that contained a gene from both species were first identified. Next the subset of groups were selected that contained only one gene from both *S. cerevisiae *and *C. elegans*, these were the genes without paralogues for both species. Then, for the *C. elegans *genes in these groups the number of genes with lethal phenotypes was calculated and expressed as a percent of the total number of *C. elegans *genes with orthologues in *S. cerevisiae *(numbers of genes are given in Additional File 1: Table S2).

To analyze the frequency of essentiality among genes with *essential *orthologues in *C. elegans*, for example, to examine how well the presence of an essential orthologue in *S. cerevisiae *predicts essentiality in *C. elegans*, the OrthoMCL groups that contained a gene from both species were first identified. Next the subset of groups were selected that contained only one gene from both *S. cerevisiae *and *C. elegans*, this was followed by identifying the subset of groups that had an essential *S. cerevisiae *orthologue. Then for the *C. elegans *genes in these groups the number of genes with lethal phenotypes was calculated and expressed as a percent of the total number of *C. elegans *genes with an essential orthologue in *S. cerevisiae *(numbers of genes are given in Additional File 1: Table S3).

The set of *C. elegans *genes without mammalian orthologues were those genes that were either in an OrthoMCL group that did not contain a gene from any of the three mammalian species in OrthoMCL (*Homo sapiens, M. musculus *or *Rattus norvegicus*) or *C. elegans *genes that were assigned to 'no_group' by OrthoMCL.

### Prediction of drug targets for parasitic nematodes

All available ESTs for *H. contortus *and *A. caninum *were downloaded from the National Center for Biotechnology Information (NCBI) and comprised 21,975 and 80,551 ESTs respectively. The ESTs were cleaned of vector using SeqClean, repeats were masked with Repeatmasker, using default parameters for both programs. We initially used the CAP3 program for clustering the ESTs, however, manual inspection of the results led us to discover that we were getting incorrect clustering - clustering of ESTs from similar but not identical genes into the same contig for example ESTs from large gene families such as collagens, lectins, cysteine proteases. We tried increasing the percent identity cutoff of the CAP3 program from the default 80% to 95% however we still found incorrect clustering on manual inspection. The ESTs were therefore individually mapped to OrthoMCL orthology groups using the initial steps of the OrthoSelect method described by Schreiber and colleagues [48]. Briefly, ESTs assigned to an orthology group had to have a sufficient BLASTX e-value, when averaged over the best hit from each species, where the default score cutoff was used. As ESTs frequently represent small fragments of genes, a caveat for this type of analysis is that the shorter length makes assignment to orthologue groups less robust.

An OrthoMCL group was defined as containing paralogues in the parasitic nematodes if any two of the ESTs had a TBLASTX hit (e-value < 1e^-10^) to each other, as well an imperfect BLASTN hit (identity < 95%). This conservative e-value was used to avoid missing potential paralogous relationships at the cost of flagging potential false positive paralogues pairs that could be manually checked. Additionally, OrthoMCL groups containing two or more *C. elegans *proteins were considered as containing paralogues. The *C. elegans *gene(s) in that group were then classed as orthologues of the parasite EST and our drug target selection criteria were applied to these *C. elegans *genes. A caveat to this OrthoSelect/OrthoMCL clustering approach is that members of a recently diverged gene family, or some genes that share only a common domain, may be classified here as paralogues, even where they now have distinct roles that are not functionally redundant. This should be improved by a preliminary EST-clustering step, which could be included for future analyses of EST datasets more amenable to such clustering. The *A. caninum *ESTs could be assigned to 2313 OrthoMCL groups, resulting in 2743 *C. elegans *genes being predicted as orthologues of the *A. caninum *ESTs. The *H. contortus *ESTs could be assigned to 2230 OrthoMCL groups, resulting in 2650 *C. elegans *genes being predicted as orthologues of the *H. contortus *ESTs.

For the assayability requirement enzymes, ion channels and GPCRs were identified using gene ontology (GO) terms. GO terms for *C. elegans *genes were obtained from the Amigo CVS database, using version 1.127 of gene_association.wb.gz and included in the database (retrieved Feb 2009 from http://cvsweb.geneontology.org/cgi-bin/cvsweb.cgi/). A custom software library, named GORuby, was developed to classify *C. elegans *genes as enzymes if they had a GO identifier for catalytic activity (GO:0003824) or one of its more specific subterms (e.g., GO:0016301 kinase activity). Similarly, a script was written to identify ion channels and GPCRs - genes that had a GO identifier for ion channel activity (GO:0005216) or G-protein coupled receptor activity (GO:0004930) or one of their more specific subterms (e.g., GO:0001584 rhodopsin-like receptor activity). The software was written in Ruby http://www.ruby-lang.org/ using the RSRuby library http://rubyforge.org/projects/rsruby/ to communicate with R http://www.r-project.org and the GO.db Bioconductor package http://www.bioconductor.org/packages/data/annotation/html/GO.db.html. Using this approach, the reference list of GO terms did not need to be loaded into a database but were instead installed with GO.db. The software is available under GPLv3 license at http://github.com/wwood/goruby.

For the *C. elegans *genes that were predicted orthologues of the *A. caninum *and *H. contortus *ESTs we identified those that met the assayability requirement, followed by identification of those we identified those that were essential in *C. elegans*. This resulted in 421 *C. elegans *genes in our final drug target list, 163 were orthologues common to both *A. caninum *and *H. contortus*, 124 were only present in *H. contortus *and 134 were only present in the larger dataset of *A. caninum*. All genes in the list were enzymes or ion channels as no GPCRs met all our selection criteria. A quantitative weighting scheme was applied according to the present analysis of the relative importance of each feature in determining essentiality - absence of paralogues, presence of orthologue (with no paralogues) in *D. melanogaster M. musculus *and/or *S. cerevisiae*, presence of essential orthologue (with no paralogues) in *D. melanogaster, M. musculus *and/or *S. cerevisiae*.

Network connectivity data for *C. elegans *genes were obtained from the Wormnet v1 (available at http://www.functionalnet.org/wormnet/); [37] and integrated into the database by mapping the genes in Wormnet to the *C. elegans *OrthoMCL genes using the gene identifiers from the Wormbase gff3 file. The "core" higher confidence set were those with linkages with LLS of ≥ 0.405465108108 (ln1.5). For each *C. elegans *gene the LLS for all linkages in the core network were added and this resulted in 10,041 genes with a network score greater than zero. This approach for the identification of drug targets in parasitic nematodes is summarised in Figure 4.

## Abbreviations

GPCR: G-protein coupled receptor; EST: expressed sequence tag; GO: Gene Ontology; LLS: log-likelihood score.

## Authors' contributions

Project was conceived by SAR and RBG. MAD and BJW performed the bioinformatic experiments, BJW assisted with creation and development of the database, MAD, BJW, RSH, SAR and RBG analysed data. SAR, RBG and MAD wrote the manuscript, all authors read and approved the final manuscript.

## Supplementary Material

Additional file 1 **Numbers of genes in orthology analysis.doc**. This file shows the numbers of genes meeting each of the criteria in our orthology comparisons of essentiality. Click here for file

Additional file 2 **Fisher's exact test results for orthology analysis.xls**. This file contains the results for Fisher's exact test applied to comparisons of essentiality for the model organisms. Click here for file

Additional file 3 **Prioritized list of drug targets for *A. caninum *and *H. contortus*.xls**. The genes in this list are the *C. elegans *predicted orthologues of the *A. caninum *and *H. contortus *ESTs, as identified in the present study. Genes that correspond to validated anthelmintic drug targets are shown in bold. All the genes in this list are predicted enzymes (by GO term) and have a lethal RNAi phenotype in *C. elegans*. Targets were first prioritized by the essentiality weighting assigned from the present study, then by network connectivity score. "Present in mammals" is defined by having an orthologue in one or more of the mammalian species in the OrthoMCL 2.0 dataset (human, rat, mouse). Essential orthologue in yeast, drosophila or mouse: ✓ = orthologue (with no paralogues) associated with a lethal phenotype, ✘ = orthologue (with no paralogues) that had only non-lethal phenotypes recorded, **? **= orthologue (with no paralogues) but no phenotype information was available. Presence of paralogues in *C. elegans *was determined from the OrthoMCL grouping. Presence of paralogues in *A. caninum *and *H. contortus *was determined as described in Materials and methods. The network connectivity score is the sum of all interaction scores (log-likelihood scores) for the gene in the Wormnet core network [37]. Click here for file

Additional file 4 **Phylogenetic trees of nematode and OrthoMCL clusters**. This file contains phylogenetic trees of illustrative gene clusters that include ESTs from parasitic nematodes. Click here for file

Additional file 5 **Essential phenotypes for *C. elegans *and *M. musculus*.doc**. This file contains the phenotype descriptions for the *C. elegans *and *M. musculus *genes that were classed as essential genes in the current analysis. Click here for file
