# Supplementary material for: Drug target prediction and prioritization: using orthology to predict essentiality in parasite genomes
Source: BMC Genomics. 2010 Apr 3;11:222. doi: 10.1186/1471-2164-11-222 (PMC2867826; doi:10.1186/1471-2164-11-222)
Supplement: Additional file 1 — Numbers of genes in orthology analysis.doc. This file shows the numbers of genes meeting each of the criteria in our orthology comparisons of essentiality. [file 1471-2164-11-222-S1.DOC]

Additional file 1:

Table 1: **Effect of paralogy on probability of essentiality of genes.**

This table shows the probability of essentiality for genes with and without paralogues. Presence of paralogues means more than one gene from the species is present in an OrthoMCL group. Percentages are numbers of genes with lethal phenotypes expressed as a fraction of genes with available phenotype information for each species and the numbers of genes are given in brackets.

|  | ***Genes with and without paralogues*** | ***Without paralogues*** | ***With paralogues*** | ***fold difference for with and without paralogues*** |
| --- | --- | --- | --- | --- |
| *C. elegans* | 16% (2941/18860 genes) | 18% (2285/12872 genes) | 11% (656/5988 genes) | +1.6-fold |
| *S. cerevisiae* | 19% (1122/5908 genes) | 23% (1050/4633 genes) | 6% (72/1275 genes) | +3.8-fold |
| *D. melanogaster* | 22% (2343/10572 genes) | 24% (1830/7641 genes) | 18% (513/2931 genes) | +1.4-fold |
| *M. musculus* | 43% (2136/5008 genes) | 40% (1214/3019 genes) | 46% (922/1989 genes) | -0.9-fold |

Table 2: **Effect of gene conservation on probability of essentiality of genes.**

This table shows the probability of essentiality for the four model organisms *C. elegans* (1), *S. cerevisiae* (2), *D. melanogaster*(3), *M. musculus*(4). The percentages are the number of genes with lethal phenotypes divided by the total no. of genes with orthologues in the species being compared and the numbers of genes are given in brackets. Essentiality was assessed for genes without paralogues.

***1)C. elegans***:

| ***C. elegans* genes with orthologues in:** | ***Probability of essentiality of C. elegans genes*** |
| --- | --- |
| *M. musculus* | 35% (958/2729 genes) |
| *D. melanogaster* | 38% (1272/3337 genes) |
| *S. cerevisiae* | 51% (770/1502 genes) |
| *D. melanogaster* + *M. musculus* | 39% (848/2184 genes) |
| *D. melanogaster* + *S. cerevisiae* | 53% (685/1298 genes) |
| *M. musculus* + *S. cerevisiae* | 52% (562/1087 genes) |
| *D. melanogaster* + *M. musculus* + *S. cerevisiae* | 53% (518/984 genes) |

***2)S. cerevisiae***:

| ***S. cerevisiae* genes with orthologues in:** | ***Probability of essentiality of S. cerevisiae genes*** |
| --- | --- |
| *M. musculus* | 41% (563/1387 genes) |
| *D. melanogaster* | 41% (647/1587 genes) |
| *C. elegans* | 42% (631/1511 genes) |
| *D. melanogaster* + *M. musculus* | 44% (521/1173 genes) |
| *D. melanogaster* + *C. elegans* | 44% (569/1303 genes) |
| *M. musculus* + *C. elegans* | 45% (490/1096 genes) |
| *D. melanogaster* + *M. musculus* + *C. elegans* | 47% (463/992 genes) |

***3)D. melanogaster***:

| ***D. melanogaster* genes with orthologues in:** | ***Probability of essentiality of D. melanogaster genes*** |
| --- | --- |
| *M. musculus* | 30% (889/2966 genes) |
| *S. cerevisiae* | 44% (597/1363 genes) |
| *C. elegans* | 35% (983/2822 genes) |
| *S. cerevisiae* + *M. musculus* | 43% (428/996 genes) |
| *S. cerevisiae* + *C. elegans* | 47% (526/1119 genes) |
| *M. musculus* + *C. elegans* | 34% (636/1856 genes) |
| *S. cerevisiae* + *M. musculus* + *C. elegans* | 45% (382/843 genes) |

***4)M. musculus***:

| ***M. musculus* genes with orthologues in:** | ***Probability of essentiality of M. musculus genes*** |
| --- | --- |
| *D. melanogaster* | 64% (498/775 genes) |
| *S. cerevisiae* | 72% (165/229 genes) |
| *C. elegans* | 66% (374/568 genes) |
| *S. cerevisiae* + *D. melanogaster* | 78% (142/183 genes) |
| *S. cerevisiae* + *C. elegans* | 77% (127/164 genes) |
| *D. melanogaster* + *C. elegans* | 70% (300/430 genes) |
| *S. cerevisiae* + *D. melanogaster* + *C. elegans* | 80% (116/145 genes) |

Table 3:

Predictions of essentiality from essential orthologues for the four model organisms *C. elegans* (1), *S. cerevisiae* (2), *D. melanogaster*(3), *M. musculus*(4). The percentages are the number of genes with lethal phenotypes divided by the total no. of genes for that criteria, the numbers of genes are given in brackets. Essentiality was assessed for genes without paralogues.

***1)C. elegans***:

| ***C. elegans* genes with orthologue with LETHAL phenotype in:** | ***Probability of essentiality of C. elegans genes*** |
| --- | --- |
| *M. musculus* | 35% (129/366 genes) |
| *D. melanogaster* | 60% (582/965 genes) |
| *S. cerevisiae* | 72% (450/622 genes) |
| *D. melanogaster* + *M. musculus* | 51% (37/73 genes) |
| *D. melanogaster* + *S. cerevisiae* | 81% (258/320 genes) |
| *M. musculus* + *S. cerevisiae* | 69% (27/39 genes) |
| *D. melanogaster* + *M. musculus* + *S. cerevisiae* | 75% (12/16 genes) |

***2)S. cerevisiae***:

| ***S. cerevisiae* genes with orthologue with LETHAL phenotype in:** | ***Probability of essentiality of S. cerevisiae genes*** |
| --- | --- |
| *M. musculus* | 30% (49/162 genes) |
| *D. melanogaster* | 60% (353/588 genes) |
| *C. elegans* | 59% (450/759 genes) |
| *D. melanogaster* + *M. musculus* | 51% (23/45 genes) |
| *D. melanogaster* + *C. elegans* | 71% (258/362 genes) |
| *M. musculus* + *C. elegans* | 39% (27/69 genes) |
| *D. melanogaster* + *M. musculus* + *C. elegans* | 48% (12/25 genes) |

***3)D. melanogaster***:

| ***D. melanogaster* genes with orthologue with LETHAL phenotype in:** | ***Probability of essentiality of D. melanogaster genes*** |
| --- | --- |
| *M. musculus* | 31% (125/406 genes) |
| *S. cerevisiae* | 64% (353/550 genes) |
| *C. elegans* | 54% (582/1080 genes) |
| *S. cerevisiae* + *M. musculus* | 66% (23/35 genes) |
| *S. cerevisiae* + *C. elegans* | 73% (258/354 genes) |
| *M. musculus* + *C. elegans* | 40% (37/92 genes) |
| *S. cerevisiae* + *M. musculus* + *C. elegans* | 57% (12/21 genes) |

***4)M. musculus***:

| ***M. musculus* genes with orthologue with LETHAL phenotype in:** | ***Probability of essentiality of M. musculus genes*** |
| --- | --- |
| *D. melanogaster* | 71% (125/177 genes) |
| *S. cerevisiae* | 94% (49/52 genes) |
| *C. elegans* | 81% (129/160 genes) |
| *S. cerevisiae* + *D. melanogaster* | 96% (23/24 genes) |
| *S. cerevisiae* + *C. elegans* | 93% (27/29 genes) |
| *D. melanogaster* + *C. elegans* | 86% (37/43 genes) |
| *S. cerevisiae* + *D. melanogaster* + *C. elegans* | 92% (12/13 genes) |
