# Supplementary material for: Drug target prediction and prioritization: using orthology to predict essentiality in parasite genomes
Source: BMC Genomics. 2010 Apr 3;11:222. doi: 10.1186/1471-2164-11-222 (PMC2867826; doi:10.1186/1471-2164-11-222)
Supplement: Additional file 4 — Phylogenetic trees of nematode and OrthoMCL clusters. This file contains phylogenetic trees of illustrative gene clusters that include ESTs from parasitic nematodes. [file 1471-2164-11-222-S4.PDF]

Additional file 4. Phylogenetic trees of nematode and OrthoMCL clusters

Three different phylogenetic trees are shown as examples of the clusters that were used to define orthology and paralogy for the drug target prediction. A and B show clusters that are defined as possessing paralogues in nematodes due to the presence of multiple genes from *C. elegans* or from one of the parasitic helminthes.

ESTs that match to each of the three representative OrthoMCL groups were assembled using CAP3 version date 04/15/05 using default parameters [1]. Assemblies were then manually inspected to avoid assembly errors. Translations were obtained by querying the assembled contigs and/or singlets against the corresponding OrthoMCL group using BLAST 2.2.17, and then inspecting the hit against the *C. elegans* orthologue. Multiple sequence alignments were found using MAFFT 6.704b linsi algorithm [2, 3] and manually cropped using JalView [4]

Distance, parsimony and maximum likelihood tree making methods were then used to infer trees using PHYLIP PROTDIST, PROTPARS [5], and PhyML [6] respectively, automated using custom-built BioRuby scripts (http://github.com/wwood/bbbin/tree/ampkb_publication). 1000 bootstrap replicates were performed for each methods.

1. Huang X, Madan A: **CAP3: A DNA sequence assembly program**. *Genome Res* 1999, **9**(9):868-877.
2. Katoh K, Misawa K, Kuma K, Miyata T: **MAFFT: a novel method for rapid multiple sequence alignment based on fast Fourier transform**. *Nucleic Acids Res* 2002, **30**(14):3059-3066.
3. Katoh K, Toh H: **Recent developments in the MAFFT multiple sequence alignment program**. *Brief Bioinform* 2008, **9**(4):286-298.
4. Clamp M, Cuff J, Searle SM, Barton GJ: **The Jalview Java alignment editor**. *Bioinformatics* 2004, **20**(3):426-427.
5. Felsenstein J: **PHYLIP - Phylogeny Inference Package (Version 3.2)**. *Cladistics* 1989, **5**:164-166. .
6. Guindon S, Gascuel O: **A simple, fast, and accurate algorithm to estimate large phylogenies by maximum likelihood**. *Syst Biol* 2003, **52**(5):696-704.

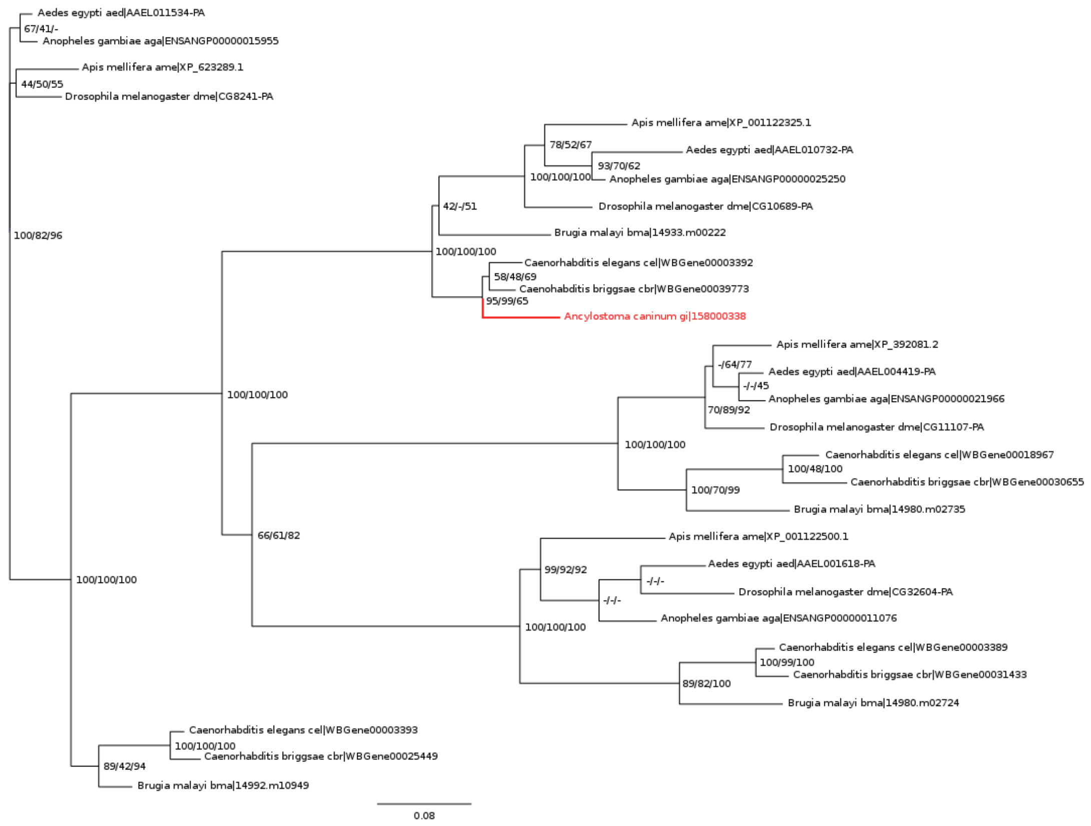

Figure A. OrthoMCL group OG2_70665 contains three *C. elegans* genes. Two *A. caninum* ESTs were assigned to it and one of these (GenBank gi: 158000338) is shown in the phylogenetic tree. Since there are multiple *C. elegans* genes in this OrthoMCL group, the likelihood of the *A. caninum* gene being essential is reduced. Figures shown beside nodes correspond to the distance, parsimony and maximum likelihood bootstrap values respectively.

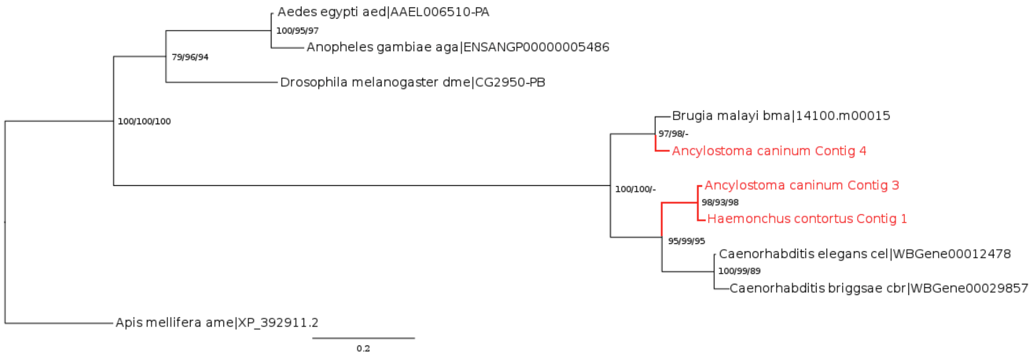

Figure B. OrthoMCL group OG2_88431 contains one *C. elegans* gene. Several ESTs from both *A. caninum* and *H. contortus* were assigned to it and two *A. caninum* and one *H. contortus* contigs are shown in the phylogenetic tree. The two *A. caninum* contigs appear to represent paralogues, and as such, are less likely to be essential genes. Figures shown beside nodes correspond to the distance, parsimony and maximum likelihood bootstrap values respectively.

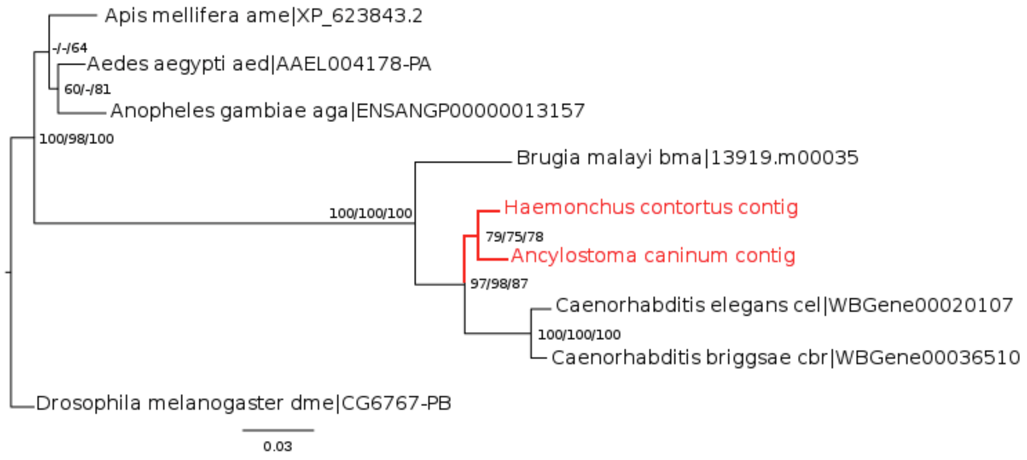

Figure C. OrthoMCL group OG2_70837 contains one *C. elegans* gene, as well as one contig each from *A. caninum* and *H. contortus*. There is no evidence of paralogy within the OrthoMCL group or contigs, and as such, this gene is more likely to be essential. Figures shown beside nodes correspond to the distance, parsimony and maximum likelihood bootstrap values respectively.
