# Supplementary material for: Drug target prediction and prioritization: using orthology to predict essentiality in parasite genomes
Source: BMC Genomics. 2010 Apr 3;11:222. doi: 10.1186/1471-2164-11-222 (PMC2867826; doi:10.1186/1471-2164-11-222)
Supplement: Additional file 5 — Essential phenotypes for C. elegans and M. musculus.doc. This file contains the phenotype descriptions for the C. elegans and M. musculus genes that were classed as essential genes in the current analysis. [file 1471-2164-11-222-S5.DOC]

**Additional File 5: Essential phenotypes for *C. elegans* and *M. musculus***

Phenotypes included in essentiality analysis were those containing the term 'lethal' for *C. elegans* and *M. musculus*:

***C. elegans***

ADL adult_lethal

EMB early_embryonic_lethal

EMB embryonic_lethal

EMB embryonic_lethal_late_emb

LET lethal

LET,LVL L2_lethal

LVL early_larval_lethal

LVL larval_lethal

LVL late_larval_lethal

LVL rod_like_larval_lethal

MEL maternal_effect_lethal_emb

XX_lethal

***M. musculus***

embryonic lethality

embryonic lethality at implantation

embryonic lethality before implantation

embryonic lethality before somite formation

embryonic lethality before turning of embryo

embryonic lethality during organogenesis

lethality at weaning

lethality throughout fetal growth and development

lethality-postnatal

lethality-prenatal/perinatal

neonatal lethality

perinatal lethality

postnatal lethality

prenatal lethality
